# Molecular organization and chromosomal localization of 5S rDNA in Amazonian *Engystomops *(Anura, Leiuperidae)

**DOI:** 10.1186/1471-2156-13-17

**Published:** 2012-03-20

**Authors:** Débora Silva Rodrigues, Miryan Rivera, Luciana Bolsoni Lourenço

**Affiliations:** 1 Department of Structural and Functional Biology, Institute of Biology, University of Campinas (UNICAMP), Campinas, SP 13083-863, Brazil; 2 Escuela de Ciencias Biológicas, Pontifícia Universidad Católica Del Ecuador, Quito, Ecuador

**Keywords:** 5S rDNA, non-transcribed spacer, chromosome, *Engystomops*

## Abstract

**Background:**

For anurans, knowledge of 5S rDNA is scarce. For *Engystomops *species, chromosomal homeologies are difficult to recognize due to the high level of inter- and intraspecific cytogenetic variation. In an attempt to better compare the karyotypes of the Amazonian species *Engystomops freibergi *and *Engystomops petersi*, and to extend the knowledge of 5S rDNA organization in anurans, the 5S rDNA sequences of Amazonian *Engystomops *species were isolated, characterized, and mapped.

**Results:**

Two types of 5S rDNA, which were readily differentiated by their NTS (non-transcribed spacer) sizes and compositions, were isolated from specimens of *E. freibergi *from Brazil and *E. petersi *from two Ecuadorian localities (Puyo and Yasuní). In the *E. freibergi *karyotypes, the entire type I 5S rDNA repeating unit hybridized to the pericentromeric region of 3p, whereas the entire type II 5S rDNA repeating unit mapped to the distal region of 6q, suggesting a differential localization of these sequences. The type I NTS probe clearly detected the 3p pericentromeric region in the karyotypes of *E. freibergi *and *E. petersi *from Puyo and the 5p pericentromeric region in the karyotype of *E. petersi *from Yasuní, but no distal or interstitial signals were observed. Interestingly, this probe also detected many centromeric regions in the three karyotypes, suggesting the presence of a satellite DNA family derived from 5S rDNA. The type II NTS probe detected only distal 6q regions in the three karyotypes, corroborating the differential distribution of the two types of 5S rDNA.

**Conclusions:**

Because the 5S rDNA types found in *Engystomops *are related to those of *Physalaemus *with respect to their nucleotide sequences and chromosomal locations, their origin likely preceded the evolutionary divergence of these genera. In addition, our data indicated homeology between Chromosome 5 in *E. petersi *from Yasuní and Chromosomes 3 in *E. freibergi *and *E. petersi *from Puyo. In addition, the chromosomal location of the type II 5S rDNA corroborates the hypothesis that the Chromosomes 6 of *E. petersi *and *E. freibergi *are homeologous despite the great differences observed between the karyotypes of the Yasuní specimens and the others.

## Background

Ribosomal RNAs (rRNA) are molecules that combine to form the basic structures of the small and large ribosomal subunits. rRNAs are transcribed by two distinct multigene families. The 45S rDNA family synthesizes the 18S, 5.8S and 28S rRNAs, and the 5S rDNA family transcribes the 5S rRNA (as reviewed in references [1-3]). The 5S ribosomal genes are found as conserved copies of 120-bp sequences arranged *in tandem *and interspersed with variable non-transcribed spacers (NTSs) that differ in length and nucleotide composition (see reviews in [3] and [4]). Within the conserved 120-bp sequence lies an internal control region (ICR) consisting of 3 characteristic regions: the A box, an intermediate element (IE), and the C box [5]. These regions act as promoters for transcription; the A box is a general binding sequence for RNA polymerase III, and the intermediate element and the C box are interaction sites for the transcription factor TFIIIA [5]. Another characteristic typically found in the presumed-functional 5S genes is a poly-T terminator region, as initially reported by Korn and Brown [6].

Despite the large variations observed for the NTSs found in 5S rDNA, several studies have reported that conserved elements are present in these regions and that they play an important role in the regulation of 5S rRNA gene expression, similar to TATA-like sequences [3], oligonucleotide sequences [6], and the D box found in mammals [7,8]. It has also been reported that a C localized at position -1 of many previously described 5S rRNA gene sequences guarantees a correct and efficient transcription start [8].

The sequences of 5S rDNA have been used as genetic and cytogenetic markers in evolutionary studies and in the identification and comparison of species, hybrids and strains [9-13]. The 120-bp region is highly conserved even among unrelated species, making it possible to isolate the 5S rDNA repeats of several species based on the available sequence from another species that is not necessarily closely related. An interesting feature of the 5S rDNA NTS that is most likely widespread throughout all vertebrate groups is the presence of two differently sized sequences, a subject that has been extensively studied in fish (see reviews in [3,14,15]).

For anurans, knowledge combining the molecular organization of the 5S rDNA sequences and their chromosomal locations are known for *Xenopus laevis, X. borealis, X. muelleri *[16,17], n families Ascaphidae, Discoglossidae e Ranidae [13,17], *Physalaemus ephippifer *[18] and *Physalaemus cuvieri *[19]. In *Xenopus *species [20] and *P. cuvieri *[19], two types of 5S rDNA sequences were found and mapped to distinct chromosomal sites. In *Xenopus *species, the distinct types of 5S rDNA, named oocyte- and somatic types, show a differential pattern of gene expression [6,16,17,21-26]. In addition to these cases with combined molecular and chromosomal analyses, studies restricted to the nucleotide sequence of the transcribed region of the 5S rDNA have been reported for the anurans *Gastrotheca riobambae *[27], *Bufo americanus, Rana pipiens *and *Rana catesbeiana *[28].

Based on a genetic analysis of morphological data, the *Engystomops *genus was revalidated by Nascimento *et al. *[29] to allocate species previously grouped in the *Physalaemus pustulosus *group. Currently, nine species comprise the *Engystomops *genus (the source studies are cited in reference [30]), and some reproductive and genetic evidences suggest the occurrence of undescribed cryptic species among the Amazonian *Engystomops *[31-33]. The cytogenetic analyses of different populations of the Amazonian species *Engystomops petersi *and *Engystomops freibergi *revealed interesting inter- and intraspecific divergences, and the possible involvement of these cytogenetic variations in incipient speciation has been suggested [33,34]. Nevertheless, the lack of available cytogenetic markers prevents the proper identification of chromosomal homeologies in these populations, and consequently, the hypothesized rearrangements responsible for the karyotypic divergences remain unknown. In the present study, we isolated and characterized the 5S rDNA sequences of *E. freibergi *and two populations of *E. petersi *and mapped their chromosomal locations, extending the knowledge of 5S rDNA organization at the genomic and chromosomal levels in these amphibians. These data facilitate the identification of possible homeologous chromosomes among the *Engystomops*, serving as an important contribution for further studies on chromosomal divergence in anurans.

## Results

### 5S ribosomal gene characterization and molecular analysis

**Figure 1:**
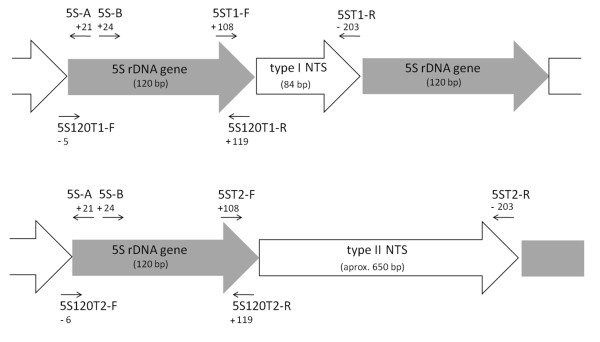
**Annealing sites of the primers used for 5S rDNA analysis**. The primers 5S-A and 5S-B were described previously by Pendás et al. [36]. For the primers sequences, see Methods section.

**Figure 2:**
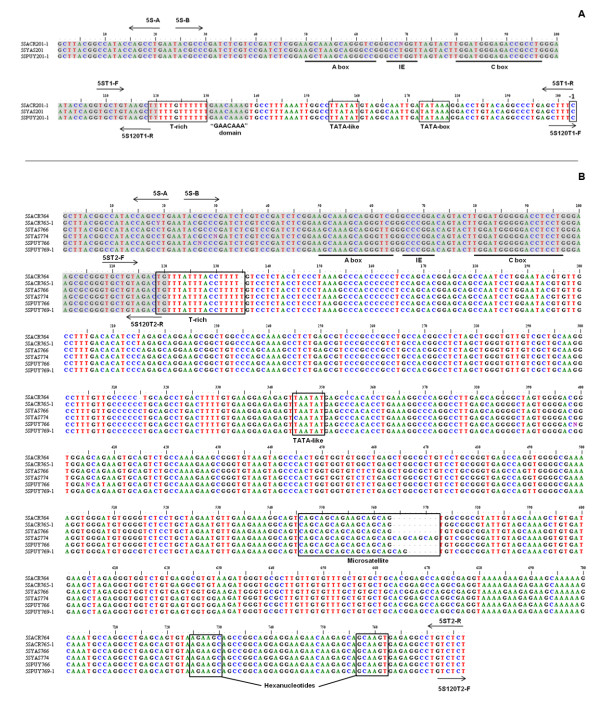
**Aligned sequences of the cloned fragments of *Engystomops freibergi *(5SACR) and *Engystomops petersi *(5SYAS and 5SPUY)**. **A**. Type I 5S rDNA sequences. **B**. Type II 5S rDNA sequences. The shaded areas indicate the presumed transcribing regions. The annealing sites of the primers used in this study are shown. Presumed regulatory elements are indicated (A box, IE, C box, poly-T, TATA box, TATA-like, hexanucleotides AGAAGC and GCAAGT, and GAACAAA sequence). The arrow in **A **points to the C nucleotide at position -1 in all the type I sequences. A microsatellite observed in type II sequences is also indicated in **B**.

PCR amplification of the segments containing the 5S rDNA from *E. freibergi *from Acre (ZUEC 9647), *E. petersi *from Yasuní (QCAZ 34948), and *E. petersi *from Puyo (QCAZ 34937) using the primers 5S-A and 5S-B (Figure 1) generated bands with fragments of approximately 750 and 200 bp in all experiments. The cloning of the *E*. *freibergi *sequences resulted in six recombinant colonies, three of which carried an insert of 201 bp, one with an insert of 764 bp, and two with inserts of 765 bp. All the cloned sequences contained a 118-bp region that corresponded to the coding sequence of the 5S ribosomal gene (Figure 2a and 2b) and NTS regions of either 84 bp (5SACR 201-1 to 3) or approximately 647 bp (5SACR 764 and 5SACR765-1 to 2). A comparison of the 84-bp fragments revealed four base substitutions among the sequences. A comparison of the 647-bp fragments showed 3 base substitutions and a deletion-insertion at position 292 (Table 1; Figure 2a and 2b).

**Table 1:** *Engystomops *type I and II 5S rDNA sequence data

Sequence name	Specimen source (Voucher number, species identification, and locality)	GenBank accession number	NTS size (bp)
5SACR201-1	ZUEC9651 - *E. freibergi*/Acre, Brazil	JF325868	84
5SACR201-2	ZUEC9651 - *E. freibergi*/Acre, Brazil	JF325869	84
5SACR201-3	ZUEC9651 - *E. freibergi*/Acre, Brazil	JF325870	84
5SYAS201	QCAZ34948 - *E. Petersi*/Yasuní, Ecuador	JF325859	84
5SPUY201-1	QCAZ34937 - *E. petersi*/Puyo, Ecuador	JF325860	84
5SPUY201-2	QCAZ34937- *E. petersi*/Puyo, Ecuador	JF325861	84
5SPUY201-3	QCAZ34937- *E. petersi*/Puyo, Ecuador	JF325862	84
5SPUY201-4	QCAZ34937- *E. petersi*/Puyo, Ecuador	JF325863	84
5SPUY201-5	QCAZ34937- *E. petersi*/Puyo, Ecuador	JF325864	84
5SPUY201-6	QCAZ34937- *E. petersi*/Puyo, Ecuador	JF325865	84
5SPUY201-7	QCAZ34937- *E. petersi*/Puyo, Ecuador	JF325866	84
5SPUY201-8	QCAZ34937- *E. petersi*/Puyo, Ecuador	JF325867	84
5SACR764	ZUEC9647 - *E. freibergi*/Acre, Brazil	JF325843	647
5SACR765-1	ZUEC9647 - *E. freibergi*/Acre, Brazil	JF325844	648
5SACR765-2	ZUEC9647 - *E. freibergi*/Acre, Brazil	JF325845	648
5SYAS766	QCAZ34948 - *E. Petersi*/Yasuní, Ecuador	JF325846	649
5SYAS774	QCAZ34948 - *E. Petersi*/Yasuní, Ecuador	JF325847	658
5SPUY766	QCAZ34937- *E. petersi*/Puyo, Ecuador	JF325848	648
5SPUY769-1	QCAZ34937- *E. petersi*/Puyo, Ecuador	JF325849	651
5SPUY769-2	QCAZ34937- *E. petersi*/Puyo, Ecuador	JF325850	651
5SPUY769-3	QCAZ34937- *E. petersi*/Puyo, Ecuador	JF325851	651
5SPUY769-4	QCAZ34937- *E. petersi*/Puyo, Ecuador	JF325852	651
5SPUY769-5	QCAZ34937- *E. petersi*/Puyo, Ecuador	JF325853	651
5SPUY769-6	QCAZ34937- *E. petersi*/Puyo, Ecuador	JF325854	651
5SPUY769-7	QCAZ34937- *E. petersi*/Puyo, Ecuador	JF325855	651
5SPUY769-8	QCAZ34937- *E. petersi*/Puyo, Ecuador	JF325856	651
5SPUY769-9	QCAZ34937- *E. petersi*/Puyo, Ecuador	JF325857	651
5SPUY769-10	QCAZ34937- *E. petersi*/Puyo, Ecuador	JF325858	651

Identification name, specimen source, GenBank accession number and NTS size of all cloned 5S rDNA sequences of *Engystomops freibergi *and *Engystomops petersi*. ZUEC: Museu de Zoologia Prof. Adão José Cardoso, at the State University of Campinas, Brazil; QCAZ: Museo de Zoología de la Pontificia Universidad Católica del Ecuador.

Three recombinant clones containing the 5S rDNA of *E. petersi *from Yasuní were recovered. The insert 5SYAS201 was shorter than the others (5SYAS766 and 5SYAS774) due to variations in their NTSs, which were 84 bp long in the insert 5SYAS201 and approximately 650 bp long in the others (Table 1). When compared with the clone 5SYAS766, clone 5SYAS774 showed nine additional nucleotides in a microsatellite DNA region (indicated at positions 546 to 554 in Figure 2b).

**Figure 3:**
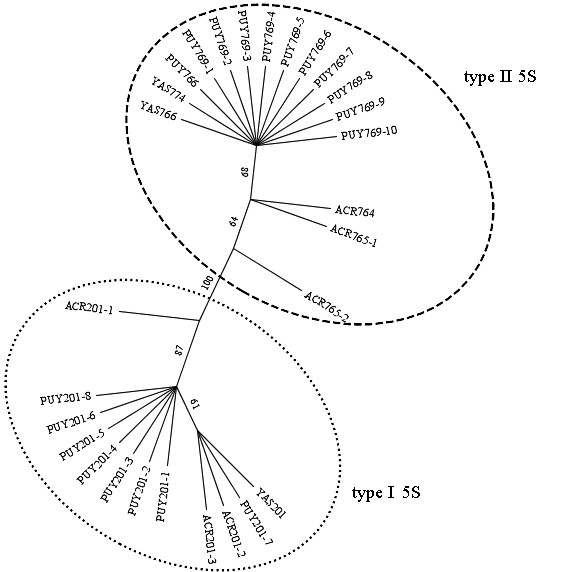
**Maximum likelihood dendrogram inferred from the coding region of the 5S rDNA sequences of *Engystomops***. The likelihood score was 265.2362. See Table **1 **for a detailed description of the sequence symbols. The dotted circle groups the type I 5S rDNA and the dashed circle groups the type II 5S rDNA sequences. Numbers above branches are bootstrap values from 1000 pseudoreplicates. Bootstrap values under 0.5 were omitted.

Nineteen recombinant clones containing the 5S rDNA of *E. petersi *from Puyo were obtained. Eleven had larger NTSs (649 bp in clone 5SPUY766 and 652 bp in clones 5SPUY769-1 to 10), whereas the remaining inserts had small NTSs of 84 bp (Figure 2). The differences in the lengths of the larger NTS segments resulted from the variation of the number of repetitions of a microsatellite DNA sequence present in these regions (positions 552 to 554 in Figure 2b). Thus, based on the NTSs, the sequences obtained can be classified into two types: type I 5S rDNA (with the small, 84-bp NTS) and type II 5S rDNA (with the large, ~650-bp NTS) (Table 1). Interestingly, the presumed 5S rDNA coding region was also characteristic for each of these types (see Table 1), as could be inferred from the maximum likelihood analysis of this specific region, in which all the type I sequences were clustered together and apart from the type II sequences group (Figure 3).

**Figure 4:**
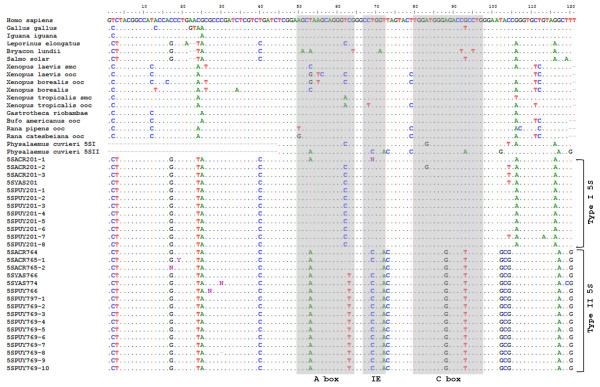
**Comparison of the 5S rDNA sequences of *Engystomops *with 5S rDNA sequences available in the GenBank**. Alignment of the presumed coding regions of the type I and II 5S rDNA sequences of *Engystomops freibergi *(Acre) and *Engystomops petersi *(Yasuní and Puyo) with the 5S rDNA sequences of other vertebrates obtained from the GenBank (accession numbers: AF250511, AF284728, AF284742, AY271269, S73107, M24954, V00647, J01009, M35055, J01010, M30904, M35176, M63899, V01425, V01426, X12622, X12623, X12624, M74438, X58365, X58368, X58367, M10817, X01309, V00589). The internal control regions are in gray (A box = positions 50 to 64; intermediate element = positions 67 to 72; C box = position 80 to 97). Note that the control regions of the *Engystomops *type I 5S rDNA sequences are more similar to those found for the other vertebrate 5S rDNA sequences than are those of the *Engystomops *type II 5S rDNA. Ooc: oocyte-type. Smc: somatic-type.

As expected, comparison of the 120 bp of the coding regions of the type I and type II 5S rDNA of *Engystomops *with those of the other anuran species and selected fish species available in GenBank revealed great similarity (Figure 4). When the type I and type II sequences of *P. cuvieri *were excluded from the analysis, there was a higher similarity between the *Engystomops *type I 5S RNA gene and the remaining 5S sequences (88-90%) than between the latter and the *Engystomops *type II gene (82%). When the *Engystomops *and *P. cuvieri *5S genes were compared, a higher similarity was observed among the sequences of the same type (Figure 4).

**Figure 5:**
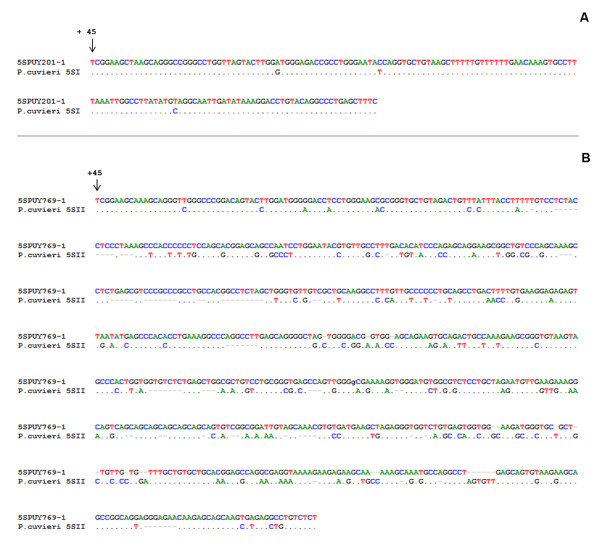
**Comparison of the 5S rDNA of *Engystomops petersi *and *Physalaemus cuvieri***. Alignment of the type I (**A**) and II (**B**) 5S rDNA of *E. petersi *from Puyo (5SPUY769-1) and of *P. cuvieri *(accession numbers: JF281131 and JF281134).

A higher similarity was also observed between the *Engystomops *and the *P. cuvieri *type I NTS (97% -100%) than between the type II NTS of the *Engystomops *species (~650 bp) and that of *P. cuvieri *(~580 bp) (90% - 92%). Because the type I NTS sequences were the same in all the *Engystomops *analyzed, and the type II NTS sequences were quite similar (average pairwise similarity: 98%; Figure 2), to better illustrate the comparison between the *Engystomops *and *P. cuvieri *NTSs, only the pairwise alignments between the sequences of single specimens of *E. petersi *from Puyo and of *P. cuvieri *are shown in Figure 5. The three elements (A box, intermediate element and C box) of the 5S gene internal control region (ICR) were identified in the presumed coding regions of all the sequences isolated from the three species of *Engystomops *(Figure 4). Interestingly, the control elements of the *Engystomops *type II sequences differed more from those available in GenBank than did the elements of the *Engystomops *type I sequences.

All sequences of the type I 5S rDNA contained the control element TATA-box in their NTS regions, which was located 25 bp upstream from the coding region. In addition, the type I sequences also had a TATA-like element located 13 nucleotides upstream from the TATA-box (Figure 2a). In the NTS of the type II sequences, an element similar to the TATA-box motif was also detected, but it was very distant from the +1 position of the presumed coding region of the 5S gene, occupying positions -345 to -350 (Figure 2b). Additionally, in the type II 5S rDNA sequences, were found two hexanucleotides (at positions -53 to -48 and -21 to -16; Figure 2b) resembling the regulatory hexanucleotides previously described for *Xenopus *[6].

Both the type I and II *Engystomops *5S sequences showed a T-rich sequence containing 4 to 5T-residues starting at position -119, which may correspond to the poly-T termination region of the 5S gene described in the literature [6,35]. Interestingly, in addition to the poly-T region, the type I sequences showed a GAACAAA sequence very similar to the sequence GAAACAA, which is found downstream from the 5S rRNA gene in fish and has been suggested to act as a terminal region [3].

**Figure 6:**
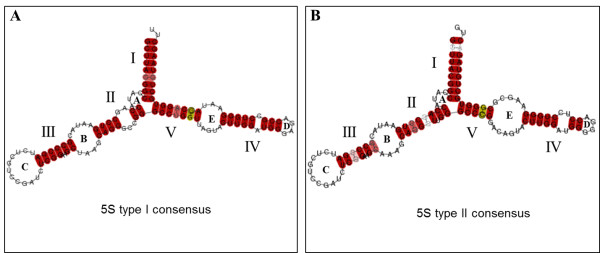
**Prediction of the 5S rRNA secondary structure of *Engystomops***. **A**. 5S rRNA of the type I consensus secondary structure. **B**. 5S rRNA of the type II consensus secondary structure.

The consensus secondary structures for the presumed type I and type II 5S rRNA is shown in Figure 6. The secondary structure of all analyzed 5S rRNAs consists of five helices (I-V), two hairpin loops (C and E), two internal loops (B and D), and a hinge region (A), arranged into the three-helix junction.

### Chromosome mapping of 5S rDNA

As expected, the karyotypes of the specimens of *E. freibergi *and *E. petersi *analyzed here were the same as those described previously by Targueta *et al. *[33]. Therefore, our study includes two of the three karyological groups recognized by Targueta *et al. *[33] among *E. petersi *specimens: the Puyo and Yasuní karyological groups. It is interesting to notice that the karyotypes of the specimens of *E. petersi *from Puyo are more similar to those of the specimens of *E. freibergi *than to the karyotype of *E. petersi *from Yasuní. To describe the mapping of the 5S rDNA sequences in these karyotypes, the previously proposed chromosome classification scheme [33] was used.

**Figure 7:**
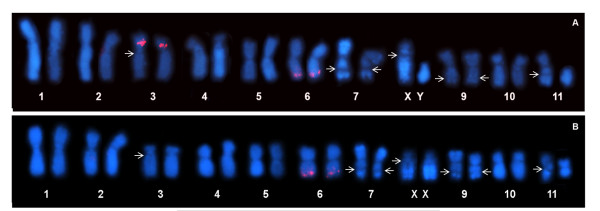
**Chromosomal mapping of the 5S and nucleolar rDNA in *Engystomops freibergi***. Karyotype of *E. freibergi *hybridized with the probe containing the entire type I 5S rDNA repeating (**A**), and with the probe containing the entire type II 5S rDNA repeating (**B**). Arrows indicate the NORs as reported by Targueta *et al. *[33] for the same specimens (A: ZUEC 14440; B: ZUEC 14458). The morphological difference between the homologues of pair 11 in **A**, and **B **results from a C-band and NOR heteromorphism, not observed in the ZUEC 14435 female whose karyotype in shown in Figure7A (for details about this heteromorphism, see [34]).

In the *E. freibergi *karyotype, a FISH probe containing the entire repeating unit of the type I 5S rDNA hybridized pericentromerically to the short arm of Chromosome 3 and also distally to the long arm of Chromosome 6 (Figure 7a). When using the probe containing the entire repeating unit of the type II 5S rDNA, only the distal region of the long arm of Chromosome 6 was detected (Figure 7b). Neither the site detected in Chromosome 3 nor the site on Chromosome 6 coincided with any nucleolar organizer regions (NORs) reported by Targueta *et al. *[33] (Figure 7).

**Figure 8:**
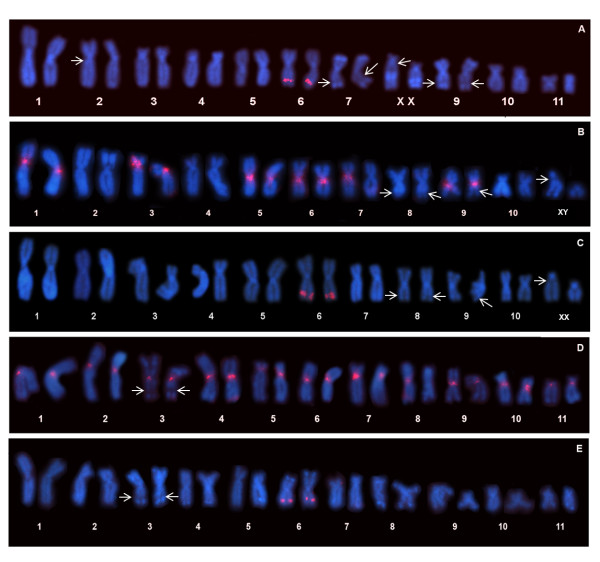
**Chromosomal mapping of the type I and II NTS in *Engystomops freibergi *and *Engystomops petersi***. Karyotypes of *E. freibergi *(**A**), *E. petersi *from Puyo (**B-C**), and *E. petersi *from Yasuní (**D-E**) hybridized with probes for type I (**B, D**) and type II (**A, C, E**) NTS. Arrows indicate secondary constrictions of the NORs. The morphological difference between the Chromosomes X in **C **is due to a heteromorphism of a terminal C-band (for details about this heteromorphism, see [33]).

Because these data suggested a differential localization of both types of 5S rDNA sequences in the *E. freibergi *karyotype, further analyses were performed using more specific probes that exclusively contained either the type I or type II NTS. In the karyotypes of *E. freibergi *and *E. petersi *from Puyo, which are morphologically very similar (for details, see [33]), the type I NTS probe detected the proximal region of the short arm of Chromosome 3 but did not detect a distal region of Chromosome 6. This probe also detected the centromeric region of several chromosomes (Figure 8b). In contrast, the type II NTS probe detected only a distal site in the long arm of Chromosome 6 in the karyotypes of both *E. freibergi *(Figure 8a) and *E. petersi *from Puyo (Figure 8c). In the karyotype of *E. petersi *from Yasuní, the type I NTS probe detected the pericentromeric region of the short arm of Chromosome 5 and the centromeric regions of several chromosomes (Figure 8d), whereas the type II NTS probe detected only a distal site on the long arm of Chromosome 6 (Figure 8e).

## Discussion

### Molecular Organization of the 5S rDNA in *Engystomops*

This study verified the occurrence of two types of 5S rDNA in the genomes of the Amazonian species of *Engystomops*, a feature widely documented in many vertebrates, including fish [10,11,36-42], the anurans *Xenopus laevis *and *Xenopus borealis *[6,16,21-24,43,44] chickens [45-47], and mammals [12,48,49]. Similarly to the findings reported in these studies, the two types of 5S rDNA sequences found in *E. freibergi *and *E. petersi *varied slightly in their corresponding coding regions, with the main difference between them found in the NTS region, which varied in length (84 bp for the type I 5S rDNA and approximately 650 bp for the type II 5S rDNA) and nucleotide composition. These two types of 5S rDNA do not, however, appear to be related to the dual system observed in *Xenopus *(i.e., the oocyte and somatic types) [6,13,16,20-22,24] because we found no similarities between the NTSs of the oocyte- or somatic-type sequences to those in the present study.

Since Ohno's publication [50], the origin of genic variants has been attributed to events of sequence duplication followed by processes that result in the divergence of the duplicated sequences. This hypothesis has been corroborated by many studies (reviewed in references [51-55]) and may also explain the presence of two types of 5S rDNA. Gene duplication may result from unequal crossing over, retropositioning, or chromosomal (or genomic) duplication [51], and the outcomes of these events are quite different, including neofunctionalization, pseudogene origin or simple preservation of gene duplicates [50,56,57].

Although our data for the Amazonian *Engystomops *do not allow us to elucidate the events involved in the origin of either type of the 5S rDNA, those events may have preceded the divergence of *Engystomops *and *Physalaemus*. Such an inference follows from the observation of higher nucleotide divergence between the sequences of the type I and type II 5S rDNAs of each species in these genera than among the sequences of the same type found in distinct species.

With respect to the functionality of the 5S rDNA sequences found in *Engystomops*, the analysis of the secondary structure of the presumed rRNAs shows that both types of sequences are consistent with the general eukaryotic 5S rRNA structure [58,59], suggesting that the type I and type II 5S rDNA sequences may have transcriptional potential. The functionality of the type I 5S rDNA sequences of *Engystomops *is corroborated by the recognition, in this type of sequences, of elements that are similar to those considered to be important for the transcriptional activity of 5S rRNA genes. Those type I 5S rDNA elements are: (i) sequences quite similar to the ICR elements; (ii) a T-rich region downstream from the presumed coding region; (iii) a TATA-box located 25 nucleotides upstream from the coding region; and (iv) the nucleotide C at position -1. In addition, in the type I 5S rDNA of *Engystomops*, the presence of a GAACAAA segment was noted, which is very similar to the sequence GAAACAA suggested to act as a terminal region of the 5S gene transcription in fish [3]. A region tentatively named the TATA-like region was also found in the *Engystomops *type I 5S rDNA, located 12 nucleotides upstream from the TATA-box. Campo *et al. *[42] reported an additional TATA-like region in the NTS regions of the 5S rDNA sequences of the fishes *Merluccius merluccius, Merluccius senegalensis*, and *Merluccius capensis*, and suggested that this TATA-box may serve as a "backup". The same hypothesis may be considered for the similar sequence found in the NTS of the type I 5S rDNA of *Engystomops*.

In contrast, some doubts remain about the transcriptional potential of the type II 5S rDNA sequences of *Engystomops*. In the type II repeats, the nucleotide at position -1 is a T and not a C; the ICR segment differed more from the ICRs of the other vertebrates used for comparison than the ICR of the type I repeats; and no TATA-box was found in the NTS. The only segment that resembles a TATA-like motif in the type II repeats of *Engystomops *was observed very distant from the region considered to be the coding region, approximately at position -420. However, a T-rich region downstream from the presumed coding region is also present in the *Engystomops *type II 5S rDNA.

It is also interesting to note that the results of previous experiments with *Xenopus *suggest that the oligonucleotides AGAAGC and AAAAGT, located at positions -28 to -23 and -18 to -13, respectively, may be involved in the initiation of 5S rDNA transcription instead of a TATA-box [6]. In the type II 5S rDNA sequences of *Engystomops*, the hexanucleotides AGAAGC and GCAAGT were found at positions -53 to -48 and -21 to -16, respectively. The similarity of these oligonucleotides to those described for *Xenopus*, despite the low similarity of the remaining NTS sequence, is an interesting issue to be considered in further analyses of the functionality of the *Engystomops *type II 5S rDNA.

### Physical Mapping of the 5S rDNA in the *Engystomops* karyotypes X evolutionary diversification in 5S rDNA

The FISH assays suggest the existence of two sites of sequence accumulation of the 5S rDNA in the karyotypes of *E. freibergi *and *E. petersi*, one on 3p (*E. freibergi *and *E. petersi *from Puyo) or 5p (*E. petersi *from Yasuní) and another on 6q. The results of these assays also suggest that the former site is exclusive to or preferentially constitutes type I sequences, whereas the latter, on 6q, is associated with type II sequences.

Ribosomal DNA repeating units are evolutionarily dynamic and appear to be able to spread throughout the genome, creating new rDNA loci [3,60-62]. The presence of two distinct 5S rDNA sequence types organized in different chromosomal regions or even on different chromosomes has been described for several fish, *e.g*., *Salmo solar *[36], *Oncorhynchus mykiss *[37], *Coregonus artedi, C. zenithicus *[63], and *Oreochromis niloticus *[40].

In anurans, the first chromosome mapping experiments for the 5S rRNA genes were conducted in *Xenopus laevis*. Using specific probes, Harper *et al. *[16] revealed a differential distribution of the two types of 5S rDNA in the *Xenopus *karyotypes, mapping the somatic-type 5S rDNA to the distal end of the long arm of Chromosome 9 in *Xenopus laevis *and *X. borealis*, and the oocyte-type to the distal ends of the majority of *Xenopus laevis *chromosomes. The authors also mapped a trace oocyte-type 5S rDNA in *X. laevis*, which is a minor class of the oocyte type, to the distal end of the long arm of Chromosome 13.

In addition to these data for the *Xenopus *karyotypes, only the 5S rDNA chromosomal sites were detected in the karyotypes of *Physalaemus ephippifer *[18] and *Physalaemus cuvieri *[19]. A probe containing the entire repeat of the type I 5S rDNA of *P. cuvieri *detected a pericentromeric region of the short arm of Chromosome 3 in both *Physalaemus *species karyotypes [18,19] whereas a probe with the entire repeat of the type II 5S rDNA of *P. cuvieri *preferentially detected a distal region of the long arm of Chromosome 6 [19]. Similarly to the above-mentioned cases, a differential localization of the two types of 5S rDNA found in the *Engystomops *species was observed in this study.

As mentioned above, taking into account the similarity of the sequences, probably the origin of the two types of 5S rDNA found in *Engystomops *and *Physalaemus *species preceded the divergence of these genera. Apparently, the origin of this dual-system involved translocation or transposition events that lead to the separation of two groups of 5S rDNA sequences, favoring the dominance of divergence forces over homogenization processes between these groups. On the other hand, the homogeneity of the 5S rDNA repeats clustered in the same chromosomal site was maintained, what may be explained by concerted evolution. As a result of these processes, two distinct types of 5S rDNA sequences, occupying different chromosomal sites, have arisen. It is worth mentioning that purifying selection may also have been involved in this scenario. In addition to concerted evolution, purifying selection has been invoked to justify the homogenization in a gene family [64,65]. Since the comparison between both types of 5S rDNA sequences of *Engystomops *showed a higher variation between the presumed coding-regions than between their NTSs, it is likely that purifying selection has been acting over these coding-regions, avoiding high level of divergence.

Another intriguing finding of this study was the hybridization of the probe that corresponds to the type I NTS to the centromeric region of various chromosomes, sites which were not detected in this analysis by the probes that potentially contain the transcribed region of the 5S rDNA. A possible explanation for this result is that the sequences associated with the centromeric regions are segments of satellite DNA derived from the 5S rDNA, a phenomenon previously reported for the fish *Hoplias malabaricus *[66] and the frog *Physalaemus cuvieri *[19].

The 5S rDNA cluster has been reported to be linked to the major rDNA sequences [4,36,37,67-69] but, in some cases, is localized to different chromosomes [18,63,70-73]. The differential chromosomal localization of the 5S and 45S rDNAs is the prevalent condition, not only in the cited examples but also in other groups, including plants [74-77].

Several authors have previously discussed the prevalence of different chromosomal sites for the 45S and 5S rDNAs over their linkage in other organisms, and a probable explanation is intrinsically related to the repetitive nature of these sequences. Others have suggested that because *tandem *repeated sequences are frequently involved in events of unequal crossing-over and gene conversion, the separation of the two great families of ribosomal DNA at different chromosomal sites would avoid disruptive interference in its organization such as undesired rearrangements between the 45S and 5S arrays [3,11].

The 5S rDNA chromosomal sites unlinked to the NORs of the *Engystomops *species represent new cytogenetic markers to be considered for their karyotypic comparison. Based on classic cytogenetic techniques, CMA_3 _and DAPI staining, and *in situ *localization of nucleolar rDNA, Targueta *et al. *[33] described the three *Engystomops *karyotypes of the present study and noted that recognizing chromosomal homeology was difficult, especially between the Yasuní karyotype and the other two. In addition, difficulties in differentiating the three very morphologically similar chromosome pairs (pairs 3, 6, and 8) in the Yasuní karyotype have been reported. In the present study, we were able to suggest a homeology between Chromosome 6 of the *E. petersi *karyotype from Yasuní and Chromosome 6 of *E. freibergi *and *E. petersi *from Puyo based on the mapping of the type II 5S rDNA sequences. Additionally, this chromosome site also constitutes a distinctive marker for Chromosome 6 in the karyotype of *E. petersi *from Yasuní, distinguishing it from Chromosome 8 and the NOR-bearing Chromosome 3. Finally, the mapping of the type I 5S rDNA sequence to Chromosome 5 of the specimens from Yasuní and Chromosome 3 of *E. freibergi *and of *E. petersi *from Puyo, which are similar chromosomes in size and morphology, may suggests that these chromosomes are homeologous.

In addition to the recognition of chromosomal homeologies among the *Engystomops *species, the 5S rDNA mapping performed here allows for a better cytogenetic comparison of *Engystomops *with its sister genus, *Physalaemus*. Chromosomes 3 of *P. cuvieri *[19] and *P. ephippifer *[18], bearing the type I 5S rDNA sequence, are morphologically similar to Chromosome 5 of *E. petersi *from Yasuní and Chromosome 3 of *E. freibergi *and *E. petersi *from Puyo. Therefore, the homeology of all these chromosomes can be strongly inferred. Similarly, we can deduce homeology among the metacentric Chromosome 6 of the *Engystomops *species and Chromosome 5 of *P. cuvieri*, which all carry the type II 5S rDNA sequences. Chromosome 5 or 6 of *P. ephippifer *likely also bears this type of sequence; however, the presence of this sequence has not been verified with a specific probe for type II 5S rDNA sequences [18]. These cytogenetic data suggest that the chromosomal sites of the 5S rDNA may be conserved in these leiuperid genera, which has not been observed for the NORs [18,33,78-84]. Therefore, the two 5S rDNA arrays appear to be independent units of evolution in *Engystomops *species, and further studies of their functionality and their relation to a possible centromeric DNA satellite sequence are necessary to provide a better understanding of the evolution of these sequences.

## Conclusions

In Amazonian *Engystomops*, two types of 5S rDNA were found and mapped to distinct chromosomal sites. Because these rDNA types are related to those found in *Physalaemus *with respect to their nucleotide sequences and chromosomal locations, their origin likely preceded the evolutionary divergence of these genera. In addition, our data revealed chromosomal homeologies among the three karyotypes of the Amazonian *Engystomops*, representing an important contribution for further studies of karyotype evolution in this genus.

## Methods

### Specimens

Specimens of *E. petersi *from Puyo (Provincia de Pastaza) and Estación Científica de Yasuní (Provincia de Olleana), located in the Ecuadorian Amazon, and specimens of *E. freibergi *from the Tejo estuary, state of Acre, Brazil were studied. The vouchers specimens are at the Museo de Zoología de la Pontificia Universidad Católica del Ecuador (QCAZ), Quito, Ecuador or at the Museu de Zoologia "Prof. Adão José Cardoso" (ZUEC) at the Universidade Estadual de Campinas (UNICAMP), Campinas, state of São Paulo, Brazil.

### Isolation and cloning of the 5S gene

Genomic DNA was isolated from liver and muscle samples stored at -80°C in the tissue collection deposited at the Laboratório de Estudos Cromossômicos em Anuros at IB-UNICAMP, Brazil. The genomic DNA isolation was performed as described [33], with a TNES solution (250 mM Tris-HCl pH 7.5, 2 M NaCl, 100 mM EDTA pH 8.0, and 2.5% SDS). After electrophoresis in 1% agarose, the DNA quality was evaluated, and its quantity was estimated. The entire repeating unit of the 5S rDNA (which includes the presumed transcribed region and the NTS) was isolated by PCR using the primers 5S-A (5'-TACGCCCGATCTCGTCCGATC-3') and 5S-B (5'-CAGGCTGGTATGGCCGTAAGC-3') [36]. The amplified fragments were purified using the GFX PCR and Gel Band DNA Purification kit (GE Healthcare - Little Chalfont, Buckinghamsire, UK) and cloned into the pGEM-T vector (pGEM-T easy Vector - Promega - Madison, WI, USA) according to the manufacturer's instructions. Recombinant vectors were used to transform competent *Escherichia coli *of the JM109 strain (Fermentas), and the cloned fragments were sequenced. Repeating units of two different sizes were obtained and named type I 5S rDNA and type II 5S rDNA. Based on their nucleotide sequences, specific primers were designed to exclusively isolate the presumed 120-bp transcribed region from the type I 5S rDNA (5S120T1-F: 5'-GCTTTCGCTTACGGCCATACC-3'; 5S120T1-R: 5'-AGCTTACAGCACCTGGTATTC-3') and the type II 5S rDNA (5S120T2-F: 5'-GTCTCTGCTTACGGCCATACC-3'; 5S120T2-R: 5'-AGTCTACAGCACCCGCGCTTC-3'). Specific primers were also designed to isolate the NTS region from the type I (5ST1-F: 5'-GCTGTAAGCTTTTTGTTTTTTGAA-3'; 5ST1-R: 5'-GAAAGCTCAGGGCCTGTACAG-3') and type II (5ST2-F: 5'-GCTGTAGACCGTTTATTTACCTT-3'; 5ST2-R: 5'-AGAGACAGGCCTCTCACTTGC-3') 5S rDNA repeats. The annealing sites of the primers used in this study are indicated in Figure 1. The resulting amplified fragments were all sequenced and analyzed.

### DNA sequencing and analysis

The cloned fragments were amplified by PCR using the T7 and SP6 primers. The amplified products were purified using the GFX PCR and Gel Band DNA Purification kit (GE Healthcare - Little Chalfont, Buckinghamsire, UK) and used directly as templates in amplification reactions using the BigDye Terminator chemistry version 3.1 (Applied Biosystems - Austin, TX, USA) according to the manufacturer's recommendations. Each cloned fragment was bi-directionally sequenced in an automatic DNA sequencer. The sequences were edited using BioEdit version 7.0.1 [85] and aligned with ClustalW software. The fragments obtained were compared with other sequences available in the GenBank database [86] and in the 5S Ribosomal RNA Database [87]. All presumed coding regions of the generated 5S rDNA sequences were assembled into a data matrix, and a Maximum Likelihood (ML) analysis was conducted using the PAUP* 4.0β10 software [88] with the evolution model K80, which was selected by Modeltest 3.7 [89] for this data set. Nodal support for the ML arrangement was assessed through a non-parametric bootstrap analysis [88], with a heuristic search based on 1,000 pseudoreplicates.

### Chromosome preparations and Fluorescent *in situ *Hybridization (FISH)

Chromosome preparations were made from the intestine and testis cell suspensions obtained from the *E. freibergi *specimens ZUEC 14435, ZUEC 14439, ZUEC 14440, and ZUEC14458, and the *E. petersi *specimens QCAZ 34946, QCAZ 34947, QCAZ 34940, and QCAZ 34942. All of these cell suspensions were available at the collection of amphibian material deposited at the Laboratório de Estudos Cromossômicos em Anuros at IB-UNICAMP, Brazil, and were used previously by Targueta *et al. *[33] to describe the karyotypes of *E. freibergi *and *E. petersi*. The cell suspensions were spotted onto clean slides, and the chromosome preparations were hybridized with the 5S rDNA fragments isolated as described above and PCR-labeled with either biotin or digoxigenin. The hybridization was performed as described elsewhere [90]. The biotin-labeled probes were detected with an anti-biotin antibody (goat anti-biotin - Vector - Burlingame CA, USA), which is recognized by a FITC-conjugated secondary antibody (anti-goat IgG-FITC - Vector - Burlingame CA, USA). The digoxigenin-labeled probes were detected with an anti-digoxigenin antibody conjugated with rhodamine. The chromosomes were counterstained with DAPI (0.5 μg/mL).

## Abbreviations

rDNA: ribosomal DNA; NTS: non-transcribed spacer; DAPI: 4 6-diamidino-2-phenylindole; 3p: short arm of Chromosome 3; 5p: short arm of Chromosome 5; 6q: long arm of Chromosome 6.

## Competing interests

The authors declare that they have no competing interests.

## Authors' contributions

DSR acquired the data and drafted the manuscript. MR helped to collect the specimens and obtain the chromosome preparations and revised the manuscript. LBL designed and coordinated the study and helped draft the manuscript. All authors read and approved the final manuscript.
